# Fish as sentinels of antimicrobial resistant bacteria, epidemic carbapenemase genes, and antibiotics in surface water

**DOI:** 10.1371/journal.pone.0272806

**Published:** 2022-09-02

**Authors:** Gregory A. Ballash, Anca Baesu, Seungjun Lee, Molly C. Mills, Dixie F. Mollenkopf, S. Mažeika P. Sullivan, Jiyoung Lee, Stephen Bayen, Thomas E. Wittum

**Affiliations:** 1 Department of Veterinary Preventive Medicine, The Ohio State University, Columbus, Ohio, United States of America; 2 Department of Food Science and Agricultural Chemistry, McGill University, Ste-Anne-de-Bellevue, Quebec, Canada; 3 Division of Environmental Health Sciences, College of Public Health, The Ohio State University, Columbus, Ohio, United States of America; 4 Schiermeier Olentangy River Wetland Research Park, School of Environment and Natural Resources, College of Food, Agricultural, and Environmental Sciences, The Ohio State University, Columbus, Ohio, United States of America; 5 Department of Food Science and Technology, The Ohio State University, Columbus, Ohio, United States of America; Georgia Southern University, UNITED STATES

## Abstract

Surface waters, especially those receiving wastewater flows, can disseminate antimicrobial resistant bacteria (ARB), antimicrobial resistance genes (ARG), and antibiotics. In the Scioto River of central Ohio, United States, we evaluated fishes as potential sentinels of ARB and antimicrobial contamination and investigated the influence of antimicrobial exposure on the fish intestinal resistome. Seventy-seven fish were collected from river reaches receiving inputs from two wastewater treatment plants that serve the greater Columbus Metropolitan Area. Fish were screened for the presence of cephalosporin-resistant (CeRO) and carbapenem-resistant (CRO) organisms, epidemic carbapenemase genes, and antibiotic drugs and metabolites using culture methods, droplet digital PCR, and ultra-high performance liquid chromatography tandem mass spectroscopy (UHPLC-MS/MS). Nearly 21% of fish harbored a CeRO in their resistome, with 19.4% exhibiting bacteria expressing an AmpC genotype encoded by *bla*_CMY_, and 7.7% with bacteria expressing an extended-spectrum β-lactamase phenotype encoded by *bla*_CTX-M._ *bla*_KPC_ and *bla*_NDM_ were present in 87.7% (57/65) and 80.4% (37/46) of the intestinal samples at an average abundance of 10^4^ copies. Three antibiotics–lincomycin (19.5%), azithromycin (31.2%) and sulfamethoxazole (3.9%)–were found in hepatic samples at average concentrations between 25–31 ng/g. Fish harboring *bla*_CTX-M_ and those exposed to azithromycin were at greater odds of being downstream of a wastewater treatment plant. Fish that bioconcentrated antibiotics in their liver were not at greater odds of harboring CeRO, CRO, or epidemic carbapenemase gene copies in their resistome. Our findings confirm that fishes can be effective bioindicators of surface waters contaminated with ARB, ARG, and antibiotics. Moreover, our findings highlight the varying importance of different mechanisms that facilitate establishment of ARB in aquatic ecosystems.

## Introduction

Antibiotic resistant bacteria (ARB)–commonly referred to as “superbugs”–are routinely discharged in treated wastewater effluent into adjacent rivers and other surface waters [1]. In particular, rivers and other aquatic ecosystems that receive wastewater inputs from hospitals and other health facilities are transport reservoirs for ARB, antimicrobial resistance genes (ARG) and antimicrobials and their metabolites [2–4]. Despite treatment, wastewater effluent serves as a source of both resistant bacteria and antimicrobial residues that are often discharged into surface waters [5, 6].

The environmental dissemination of ARB and ARG beyond the location of antimicrobial use represents a critical threat to public, veterinary, and environmental health. Terrestrial wild animals are known to harbor ARB and can enable the spread of ARB to other environmental compartments and hosts [7]. Aquatic animals such as otters and hippopotamus can also be ARB hosts, with aquatic vegetation and river sediments implicated as the likely source [8]. For fish, a spate of research has linked ARB to farmed fish [9, 10]. However, very few studies have tested for wild fish as potential bioindicators of ARB. Furthermore, the relative contributions of emergence pathways of ARB in the environment–either by introduction and establishment of ARB and/or ARG or via direct selection of resistant subpopulations via therapeutic and subtherapeutic concentrations of antimicrobials or their metabolites–have not been established.

Owing to the multiuse nature of rivers and other surface waters (e.g., drinking, consumptive fishing, recreational activities, agricultural irrigation, etc.), an improved understanding of potential sentinels of ARB and the dynamics underlying the establishment and persistence of ARB will be critical in developing strategies to minimize dissemination of ARB to a broad web of other environments and hosts, including humans [11]. Here, we utilized fish from a midwestern U.S. river that receives treated wastewater flows from a major metropolitan area to determine the presence of ARB and antimicrobial contamination and to understand the influence of antimicrobial exposure on the fish intestinal resistome.

## Methods

### Study site and fish sampling

**Fig 1:**
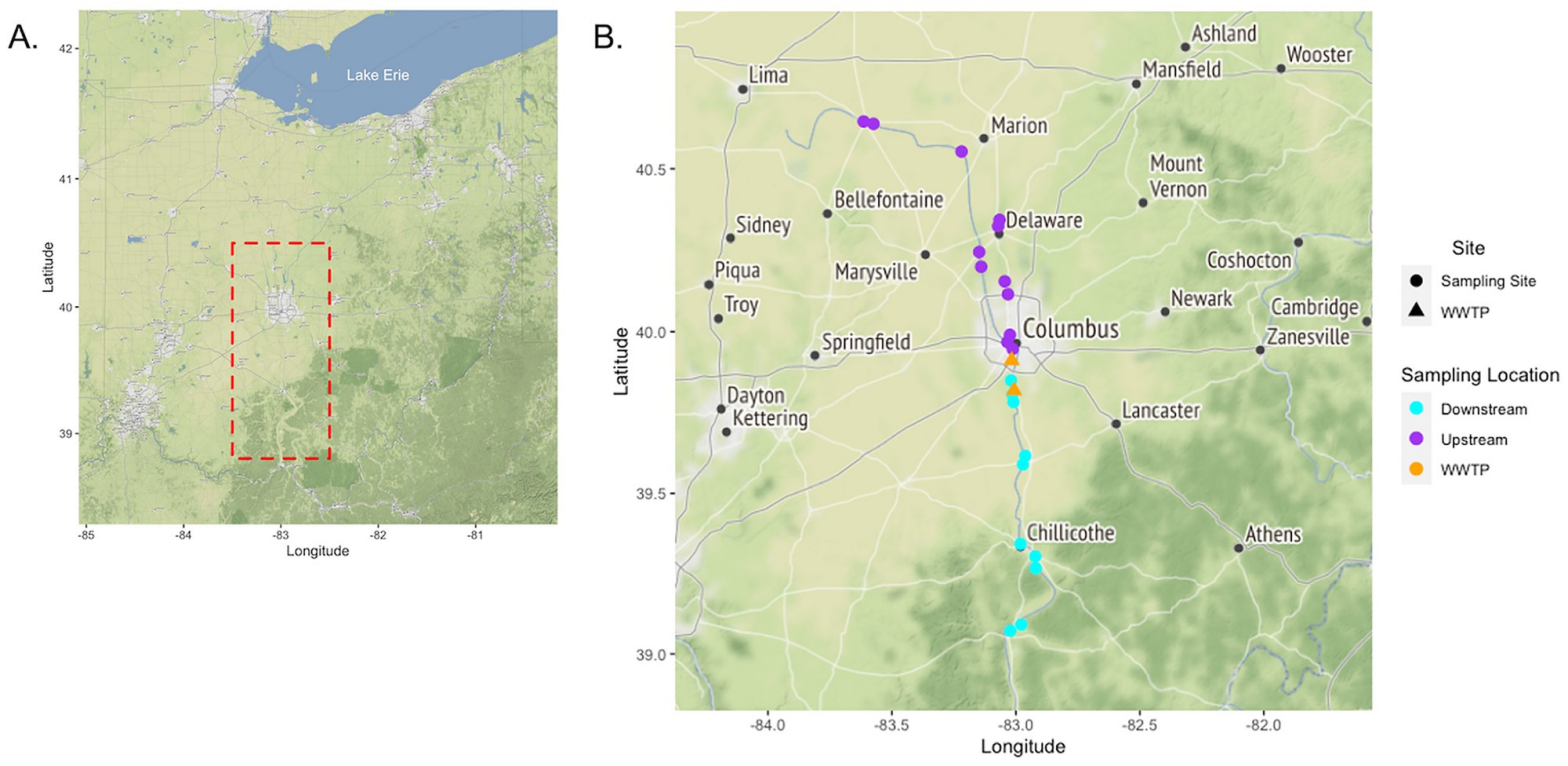
Distribution of fish sampling sites and WWTP along the Scioto River Watershed in Central Ohio. A) Map of the Ohio region. Red dash box indicates a zoomed-region of the study sample area exemplified in B. B) The study area centered in Scioto Darby Watershed in Central Ohio. Sampling sites are colored circles based on their distribution upstream or downstream of WWTP. WWTP are represented as orange triangles.

Fish were sampled from the Scioto River watershed in Columbus, Ohio during 2017 and 2018 (Fig 1). This watershed receives wastewater flows from two wastewater treatment plants that serve Columbus and surrounding suburbs. These wastewater treatment plants receive a variety of wastewater, including hospital inputs, that amounts to an average of 712 million liters of treated wastewater per day. Fish were sampled from 24 sites along the watershed using both boat and backpack electrofishers following Dorobek et al. [12]. Fish were identified to species level and transported on ice to the laboratory where hepatic and intestinal contents were collected using sterile instruments. Access to sampling sites and the collection of fish was granted via the Ohio Division of Wildlife, Wild Animal Permit-Scientific Collection 21–134. The capture, euthanasia and sampling of fish and their tissues was reviewed and approved by the Ohio State University Institutional Animal Care and Use Committee (IACUC#: 2016A00000095; IACUC#: 2009A0215-R3). Euthanasia was accomplished by submersing fish in a solution of water and buffered MS-222 (>250 mg MS-222 per liter of water) or cervical dislocation followed by pithing. Liver contents were stored in amber vials at -80°C.

### Sample processing for phenotypic and genotypic antimicrobial resistance

A swab of intestinal content was inoculated in 9 mL of MacConkey broth modified with 2 μg mL^-1^ of cefotaxime for 24 h. An inoculate of broth was streaked on three MacConkey plates in parallel–one modified with 8 μg mL^-1^ cefoxitin, one with 4 μg mL^-1^ of cefepime, and one with 0.5 μg mL^-1^ of meropenem and 70 μg mL^-1^ of zinc heptahydrate for isolation of bacteria expressing AmpC, extended-spectrum β-lactamase (ESBL), and carbapenem-resistant phenotypes, respectively. One lactose fermenting, indole positive AmpC-like and ESBL colony was tested for the presence of the *bla*_CMY_ and *bla*_CTX-M_ using previously reported PCR protocols and primers [13, 14]. Up to three carbapenem-resistant isolates with different colony morphology were tested for carbapenemase production using the CarbaNP test and speciated by MALDI-TOF [15]. The remaining intestinal contents were scrutinized for the epidemic carbapenemase genes, *bla*_KPC_ and *bla*_NDM_ using droplet digital PCR to quantitatively estimate gene copy numbers per gram of intestinal contents as previously described [1].

### Target and suspect screening of antimicrobial residues

Frozen liver samples were homogenized, and extractions were conducted on 0.5 g of sample as previously described [16]. In brief, a 100 μL sample of extract was diluted to 1 mL with water and analyzed using an Agilent UHPLC 1290 coupled with an Agilent 6545 QTOF-ESI-MS in both positive and negative ionization modes. Because fish hepatic tissue is not abundant in some species, only one replicate could be conducted. In samples where the liver weighed less than 0.5g, the whole sample was used. Sample extracts were tested for antimicrobial residues using 12 targeted antibiotic compounds (S1 Table). In addition, sample extracts were screened for suspect antimicrobial targets using the Agilent Mass Hunter Profinder software B.010.0 and importing profinder archive files (.pfa) into Mass Profiler Professional (v 15.0 Agilent Technologies) for searches among the Agilent *Veterinary Drug PCDL* (2153 compounds) and *Water Screening PCDL* (1451 compounds) libraries. The method was evaluated earlier based on recovery, precision and matrix effect, with different approaches, e.g., quality control samples, taken to assess the quality of the data [16].

### Data analysis and visualization

Logistic regression models were utilized to characterize potential associations between the presence of ARB and ARG and exposure to antibiotic compounds, site of collection (upstream vs. downstream of WWTP), and foraging group (benthic feeder vs. water-column feeder) based on species identification. Similar models were utilized to characterize exposure to antibiotic compounds to the site of collection and foraging group. In models where odds ratios could not be calculated, Fisher’s exact test was utilized to detect statistical associations. All statistical analysis and data visualization was conducted using STATA v.15.1 (StataCorp LLC, College Station, TX, USA) and GraphPad Prism v.9.0.0 (GraphPad Software, San Diego, CA, USA).

Geospatial mapping of the sample site was visualized using the “ggmap” package in R to import publicly available, open-source Stamen maps (available at https://stamen.com/open-source/). Sampling sites and wastewater treatment plant data layers were overlaid on each map using the “ggplot2” package in R.

## Results and discussion

**Fig 2:**
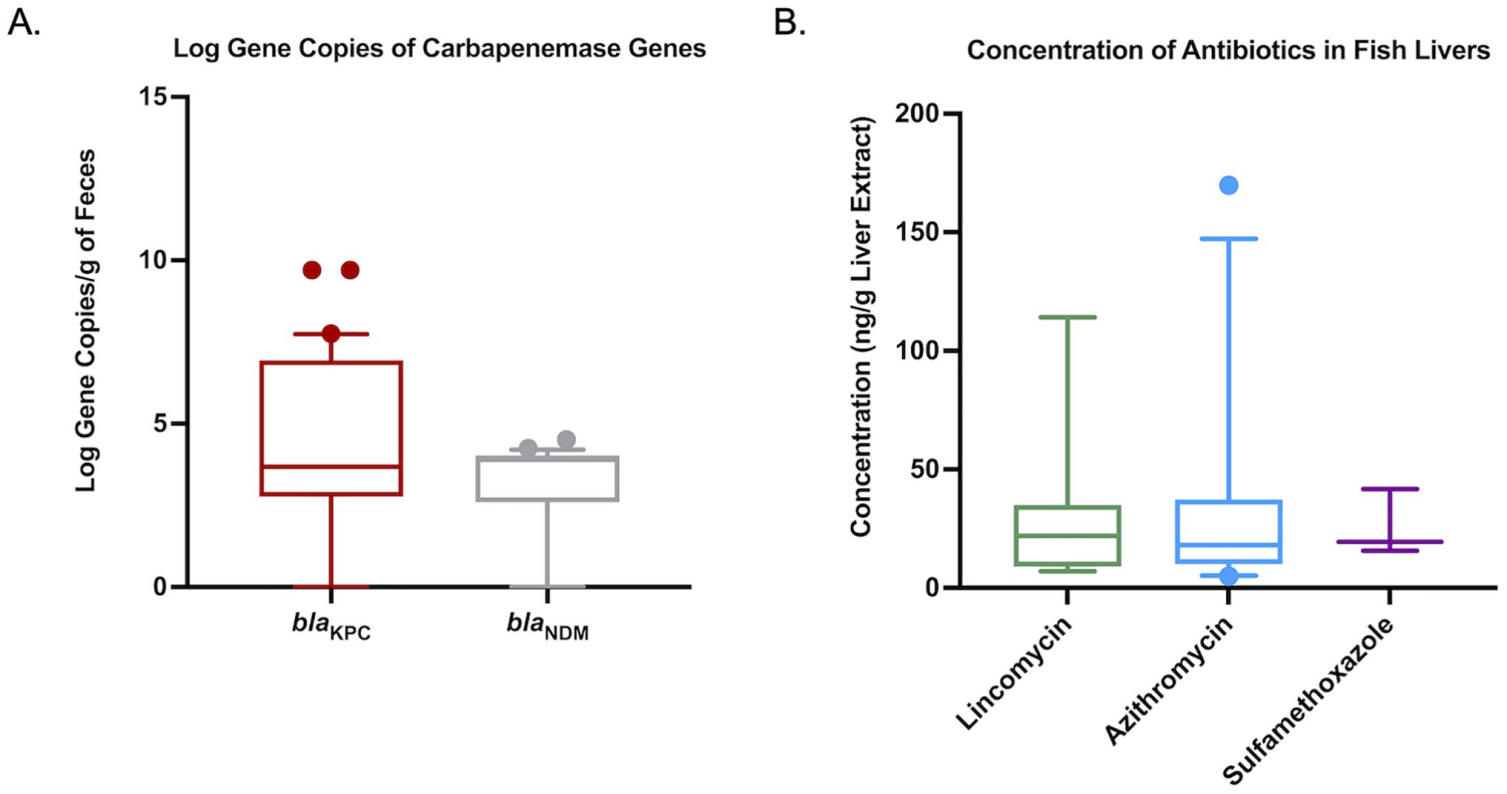
Box-and-whisker plots of concentrations of epidemic carbapenemase genes found in fish intestinal contents (A) and antibiotics (B) from liver extracts. The lower boundary of the box-and-whisker diagram indicates the 25^th^ percentile (Q1), the line within the box represents the median, and the upper boundary of the box represents the 75^th^ percentile (Q3). Error bars indicate 95^th^ and 5^th^ percentile values. Dots indicate values outside the upper and lower percentile limits.

A total of 77 fish were collected representing 22 species and both benthic and water-column feeders (S2 Table). Approximately 20% (16/77) of fish harbored an AmpC-like and/or ESBL phenotype conferred by the *bla*_CMY_ and *bla*_CTX-M_ gene, respectively (Table 1). Seven isolates produced a carbapenemase (9.1%), however these isolates represented non-epidemic strains that have species-specific chromosomally encoded carbapenemases. Of the 77 fish sampled, 65 (84.4%) and 46 (59.7%) had adequate intestinal contents to conduct ddPCR for *bla*_KPC_ and *bla*_NDM_. Fifty-seven (87.7%) samples harbored a *bla*_KPC_ gene and 37 (80.4%) harbored a *bla*_NDM_ gene in their intestinal resistome (Table 1). When combined, 100% of the 65 fish tested had either a *bla*_KPC_ or *bla*_NDM_ gene, or both. Gene abundance for both *bla*_KPC_ and *bla*_NDM_ averaged 10^4^ copies per gram of intestinal contents (Fig 2A). In general, ARB and ARG were not associated with ecological factors such as being downstream of WWTP or foraging status, although, the *bla*_CTX-M_ was more frequently identified in fish collected downstream (*p* = 0.012) (Table 2).

**Table 1:** Prevalence of fish harboring ARB and ARG in their intestinal contents and antibiotics in their hepatic extracts.

	Prevalence (%)	N
**Bacterial Phenotype**		
AmpC/CMY Phenotype	15 (19.4)	77
ESBL/CTX-M Phenotype	6 (7.7)	77
Carbapenemase-Producer	7 (9.1)	77
Total	16 (20.8)	77
**Carbapenemase-Gene**		
*bla*_KPC_	57 (87.7)	65
*bla*_NDM_	37 (80.4)	46
**Antibiotic Exposure**		
Lincomycin	15 (19.5)	77
Azithromycin	24 (31.2)	77
Sulfamethoxazole	3 (3.9)	77
Total	37 (48.1)	77

**Table 2:** Logistic regression output used to determine associations between outcomes (Antimicrobial Resistant Bacteria and Antimicrobial Resistance Genes) and predictors (Distribution around WWTP sites, foraging group and exposure to antibiotics as determined by UHPLC-MS).

	Antimicrobial Resistant Bacteria (ARB)									Antimicrobial Resistance Genes (ARG)					
	CMY (n = 77)			CTX-M (n = 77)			CPO (n = 77)			KPC (n = 65)			NDM (n = 46)		
Predictors	OR	95% CI	P-value	OR	95% CI	P-value	OR	95% CI	P-value	OR	95% CI	P-value	OR	95% CI	P-value
WWTP Site															
Upstream	Ref			Ref			Ref			Ref			Ref		
Downstream	2.43	(0.74, 7.94)	0.14	NA	NA	0.012	2.18	(0.37, 12.65)	0.39	2.43	(0.74, 7.94)	0.14	1.75	(0.31, 9.88)	0.53
Foraging Group															
Water Column	Ref			Ref			Ref			Ref			Ref		
Benthic	1.71	(0.54, 5.38)	0.36	2.18	(0.37, 12.65)	0.39	0.49	(0.08, 2.83)	0.42	1.71	(0.54, 5.38)	0.36	2.18	(0.37, 12.65)	0.39
Exposure to Antibiotics															
No Exposure	Ref			Ref			Ref			Ref			Ref		
Any Antibiotic	0.93	(0.30, 2.89)	0.91	2.3	(0.40, 13.39)	0.35	2.3	(0.40, 13.39)	0.353	0.93	(0.30, 2.89)	0.91	2.3	(0.40, 13.39)	0.35
Lincomycin	0.58	(0.12, 2.90)	0.51	0.81	(0.09, 7.54)	0.86	2.23	(0.37, 13.51)	0.38	0.58	(0.12, 2.90)	0.51	0.81	(0.09, 7.54)	0.86
Azithromycin	1.63	(0.51, 5.25)	0.41	2.92	(0.47, 18.37)	0.25	5.1	(0.86, 30.07)	0.072	1.63	(0.51, 5.25)	0.41	0.33	(0.08, 1.28)	0.14
SMZ-TMP	NA	NA	1	NA	NA	1	NA	NA	1	NA	NA	1	NA	NA	1

Predictors with “Ref” values are referent groups. Predictors with NA values for odds ratios (OR) and 95% confidence intervals (95% CI) are associations that were evaluated by Fisher’s Exact Test.

**Fig 3:**
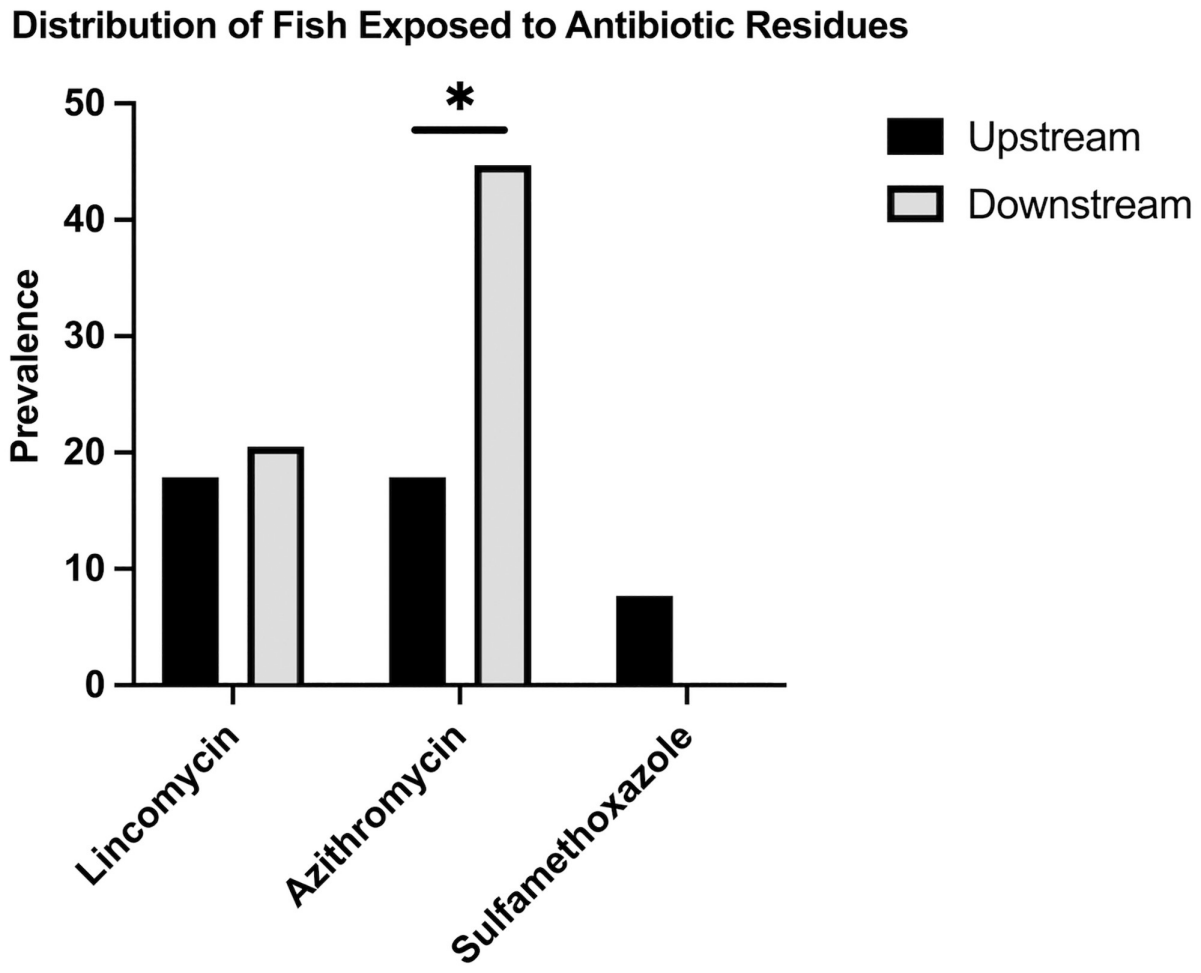
Exposure of fish to the three antimicrobials tested stratified by their presence upstream or downstream of a wastewater treatment plant. Bar with asterisk (*) indicates a statistically significant (P<0.05) association between the presence of azithromycin in the liver and where fish were sampled in relation to the wastewater treatment plants.

We identified three antibiotics representing three different drug classes that bioaccumulated within the hepatic tissue of fish (Table 1). Antimicrobial exposure, as evident by bioaccumulation of these compounds in the liver, was present in 37 (48.1%) of the fish tested. Lincomycin exposure was present in 15 (19.5%) fish livers and averaged 30.26 ng g^-1^ (Fig 2B). Azithromycin was present in 24 (31.2%) of fish livers at an average concentration of 30.6 ng g^-1^. Sulfamethoxazole was present in three (3.9%) fish livers at an average concentration of 25.6 ng g^-1^. Ecological factors did not influence antibiotic exposure, but fish downstream of a WWTP were at greater odds of being exposed to azithromycin (OR: 3.7; 95% CI: 1.31–10.45; *p* = 0.013) (Fig 3).

Some ARB and antibiotic concentrations in fish were associated with locations downstream of a WWTP, suggesting WWTP inputs may alter environmental contamination. Previous studies corroborate this conclusion as WWTP effluents can harbor ARB, ARG and antibiotics and these inputs are often more prevalent downstream of WWTP discharge points [17, 18]. However, in this study some antibiotic concentrations and ARB were not detected at a higher amount or frequency downstream of WWTP inputs. This likely reflects the importance of fish movements throughout the stream where they can spread ARB and ARG within the ecosystem’s microenvironments.

There was no association between the three antimicrobials found in hepatic extracts and the presence of ARB and ARG in the intestinal resistome. The presence of azithromycin in the liver increased the odds of harboring a *bla*_CTX-M_ isolate (OR: 5.1; 95% CI: 0.86, 30.1; P = 0.072) in fish intestinal samples (Table 2). However, after controlling for fish located downstream of a WWTP, the adjusted odds ratio was 2.92 and was not significantly associated with the presence of *bla*_CTX-M_ harboring bacteria in the gut (95% CI: 0.47; 18.37; P = 0.253).

The lack of associations between the intestinal resistome and exposure to the three antibiotics suggests that exposure to these antimicrobials does not significantly contribute to the establishment and dissemination of β-lactam resistant bacteria and genes in the environment. While β-lactams drugs are one of the most commonly prescribed antibiotics, they are inherently unstable compounds that are degraded by several biotic and abiotic factors [19, 20]. Reports of β-lactams drugs in surface water are uncommon and, if present, they are often at trace levels [21–23]. However, ARB and ARG are frequently reported in wastewater effluent and surface waters [2, 24, 25]. When our data is put in the context of previous findings, it suggests that contamination of environmental surface waters with β-lactam resistance bacteria and genes likely serves a greater role of establishing environmental and wildlife reservoirs compared to exposure/selective pressure to the antibiotics tested in this study.

## Conclusions

Overall, our findings support that fish are exposed to ARB, ARG, and antibiotics that contaminate surface waters, and they are likely reliable bioindicators of ARB in surface waters. The potential utility of fish as sentinels of ARB contamination is further supported given that they are ubiquitous, represent multiple trophic levels, integrate the abiotic environment through their foraging habitats, and have long been used as indicators of aquatic ecosystem condition [26]. Further research will be needed to investigate how diet and life-history strategies may influence ARB transmission in fish, and the potential for target taxonomic (i.e., species or families) or functional (e.g., foraging guild) groups to be the most effective sentinels of ARB contamination [27]. Fish also likely play an important role in disseminating these contaminants within surface water ecosystems, as well as into terrestrial environments via aquatic-terrestrial food-web linkages [28]. Contamination of surface water poses a hypothetical *One Health* risk as surface water is used for numerous activities important to human, animal, and environmental health.

## Supporting information

S1 Table Antibiotic drugs targeted in fish livers by tandem HPLC-MS/MS and their method detection limits and limits of quantification. (XLSX)

S2 Table Metadata from fish sampling and processing. (XLSX)
